# The Kynurenine Metabolite 3-Hydroxykynurenine is Associated With Risk of Heart Failure in Patients With Predominantly Stable Coronary Artery Disease

**DOI:** 10.1177/11786469261479281

**Published:** 2026-08-18

**Authors:** Anders Lund, Jan Erik Nordrehaug, Kjell-Christian Flo, Kyrre Hasås Toresen, Christian Alsing, Adrian McCann, Per Magne Ueland, Ottar Nygård, Lasse M. Giil

**Affiliations:** 1 Department of Anesthesiology, Haukeland University Hospital, Bergen, Norway; 2 Department of Clinical Sciences, University of Bergen, Norway; 3 Voss Hospital, Haukeland University Hospital, Bergen, Norway; 4 Department of Internal Medicine, Haraldsplass Deaconess Hospital, Bergen, Norway; 5 Bevital AS, Bergen, Norway; 6 Department of Heart Disease, Haukeland University Hospital, Bergen, Norway

**Keywords:** heart failure, incidence, kynurenine pathway, hydroxykynurenine, 3-hydroxykynurenine

## Abstract

**Background:**

Inflammation and immune activation contribute to the development and progression of heart failure (HF). The kynurenine pathway, linking tryptophan metabolism to inflammation, oxidative stress, and cell death by way of its metabolites (kynurenines), has not been studied as a pathway associated with risk for incident HF.

**Aims:**

To investigate whether kynurenine metabolites are associated with incident HF in patients with predominantly stable coronary artery disease.

**Methods:**

Serum kynurenine metabolites were quantified in 3841 patients who underwent elective coronary angiography for evaluation of chest pain. Fasting was not routine. Patients with established HF at baseline were excluded. The hazard for incident HF was estimated using Cox regression, adjusted for age, gender, current smoking, diabetes, hypertension, previous myocardial infarction, body mass index, glomerular filtration rate, troponin T, left ventricular ejection fraction, and resting heart rate.

**Results:**

During follow-up, 221 participants developed HF. Higher serum concentrations of 3-hydroxykynurenine (HK) were associated with increased HF risk (adjusted HR 1.31, 95% CI 1.07-1.46, *P* < .001).

**Conclusion:**

Higher plasma HK was independently associated with incident HF in patients with predominantly stable coronary artery disease. These findings support a possible link between kynurenine pathway activity and future HF risk, but the underlying mechanisms and clinical implications remain to be established.

## Introduction

Patients who develop heart failure (HF) frequently have classical risk factors such as age, hypertension, coronary artery disease, diabetes mellitus, chronic kidney disease, and obesity.^1^ However, these factors explain only part of the observed risk. Management is still insufficient, and HF is often detected in late stages when severe cardiac dysfunction and symptoms are already present. Therefore, prevention is a major focus of research, and thus identifying early biomarkers related to relevant pathophysiological processes is a central goal.

Studies suggest that chronic inflammation is an important and early event in the development of HF.^2^ Accordingly, multiple investigators have sought to identify inflammatory biomarkers in asymptomatic at-risk individuals.^3,4^ The main body of research has focused on cytokines and C-reactive protein (CRP). However, during inflammation there is an orchestrated shift in metabolism with induction of several rate-limiting enzymes in immune-sensitive metabolic pathways.

**Figure 1.**
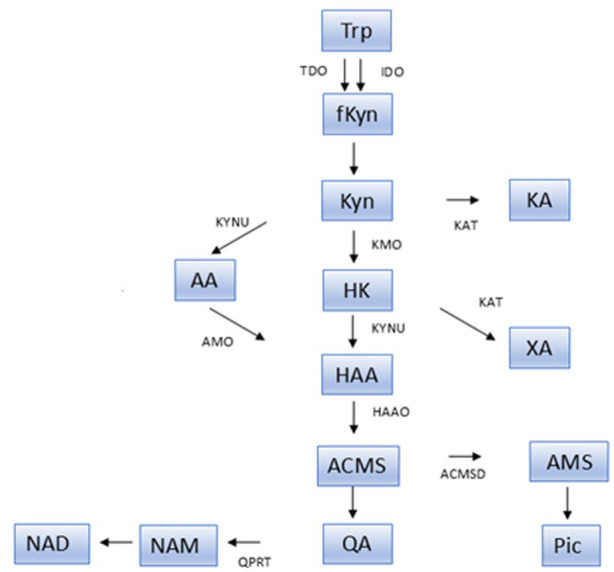
Kynurenine pathway. Tryptophan (Trp) is converted to N-formylkynurenine (fKyn) by tryptophan 2,3-dioxygenase (TDO) or indoleamine 2,3-dioxygenase (IDO), and then further to kynurenine (Kyn). From Kyn, the pathway branches toward kynurenic acid (KA), anthranilic acid (AA), and 3-hydroxykynurenine (HK). HK may be converted to xanthurenic acid (XA) or further metabolized to 3-hydroxyanthranilic acid (HAA), followed by formation of 2-amino-3-carboxymuconate-6-semialdehyde (ACMS). ACMS may either proceed toward quinolinic acid (QA), with subsequent conversion to nicotinamide adenine dinucleotide (NAD), or be diverted toward 2-aminomuconate semialdehyde (AMS) and picolinic acid (Pic). The figure illustrates the main enzymatic steps and branch points relevant to the metabolites measured in this work.

The kynurenine pathway (KP) is activated during both acute and chronic inflammation. The mechanism involves interferon-γ (IFN-γ) induction of the first and rate-limiting enzyme, indoleamine 2,3 dioxygenase (IDO), converting tryptophan (Trp) to kynurenine (Kyn)^5^ (Figure 1). In addition to the induction of IDO,^6^ downstream metabolites of the KP, collectively referred to here as kynurenines, have attracted significant interest in relation to HF since one of its intermediate metabolites, 3-hydroxykynurenine (HK), can induce apoptosis in endothelial cells.^7^ Biomarkers related to cardiomyocyte apoptosis could have significant potential, as the cardiac troponin T (TnT) level is a significant determinant of HF risk.^8^ Further, kynurenine (Kyn), generated from the amino acid tryptophan (Trp) has been shown to have vasoactive properties,^9^ and multiple metabolites of the KP, including xanthurenic acid (XA), picolinic acid (Pic), and quinolinic acid (QA), have been linked to oxidative stress and inflammation.^10,11^

We have previously shown an association between plasma levels of kynurenines, including HK, QA and XA, and mortality in patients with manifest HF,^12^ and Kyn has been associated with heart failure severity.^13^ Indeed, kynurenines are associated with both increased risk in patients with clinical symptoms of heart failure and with left ventricular remodeling.^13^ Previous clinical studies have mainly examined kynurenines in established HF or prognosis,^12,13^ while a more recent prospective nested case-control study reported associations of KTR, KA, and QA with incident HF in a high cardiovascular risk population.^14^ However, whether a broader KP profile is associated with incident HF in patients with stable CAD but without HF at baseline remains unclear. It is also uncertain whether such associations differ from those observed for incident acute myocardial infarction (AMI) within the same population.

We aimed to investigate whether KP metabolites are associated with risk of incident heart failure in patients with predominantly stable coronary artery disease. The association between kynurenines and incident AMI was provided for comparison. Further, we aimed to establish the degree of association, if any, between kynurenines, baseline TnT and left ventricular ejection fraction (LVEF).

## Materials and Methods

### Study Participants

**Figure 2.**
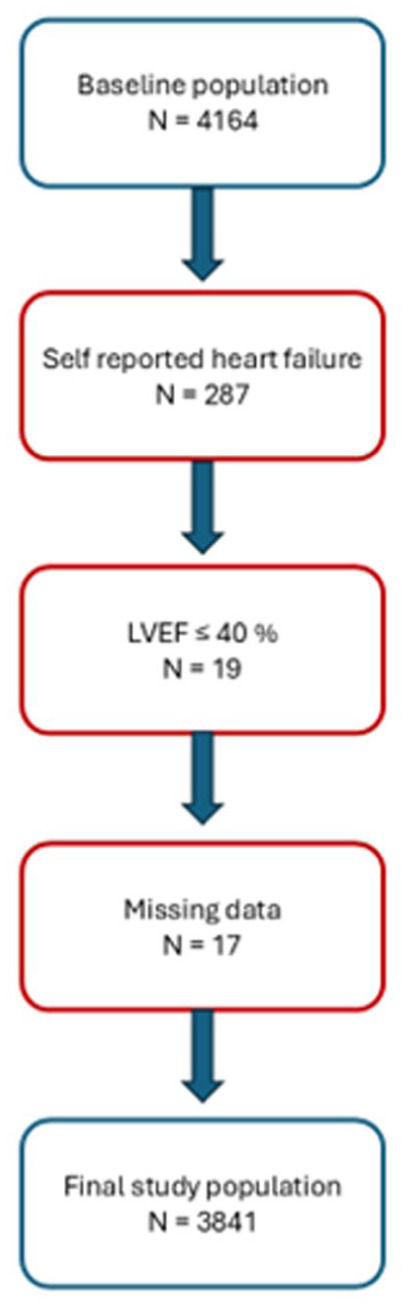
Flow diagram of participant selection. From the baseline cohort of 4164 patients who underwent coronary angiography, patients with self-reported heart failure (HF) were excluded first. An additional 19 patients with left ventricular ejection fraction (LVEF) ⩽40% on baseline cineangiography were then excluded, followed by 17 patients with missing data on either the previous selection variables or kynurenine measurements. This yielded a final study population of 3841 participants. Abbreviations: HF, heart failure; LVEF, left ventricular ejection fraction.

Study participants were recruited from the prospective Western Norway Coronary Angiography Cohort (WECAC) study from 1999 to 2004. Participants (N = 4164) underwent coronary angiography at Haukeland or Stavanger University Hospital.^15^ Patient characteristics were obtained at baseline by medical history, clinical examination, questionnaires, and a review of hospital records using standardized procedures. All patients underwent cardiac catheterization by a trained cardiologist. The severity of coronary artery disease was defined by the number (0-3) of significantly stenosed vessels, defined by lumen narrowing ⩾ 50% in the main coronary arteries. A left main stem with stenosis was classified as two-vessel disease. LVEF was determined by ventriculography. Previous myocardial infarction was registered from hospital records. Patients with self-reported HF (n = 287), an additional 19 patients with LVEF ⩽40% on baseline cineangiography, and 17 patients with missing data on these variables or kynurenine measurements were excluded, yielding a final baseline population of 3841 patients (Figure 2). The study did not include subjects with formal screening for HF. Thus, we did not measure baseline N-terminal prohormone of brain natriuretic peptide (NT-proBNP).

The diagnosis of HF after baseline was obtained through linkage to hospital records and included all cases classified as HF by clinicians according to the International Classification of Diseases, Tenth Revision (ICD-10). The diagnosis of AMI was also based on ICD-10 coding in hospital records. For the time-to-event analyses, participants were followed from baseline until the first recorded HF or AMI event, death, or the end of follow-up on 1 January 2010, whichever occurred first. As the endpoint was identified after completion of inclusion, the study represents a convenience sample.

### Ethics

This study was approved by the Regional Ethics Committee (REC) West, University of Bergen, Faculty of Medicine, Bergen, Norway (REC approval number: 2013/2022, a subproject of the study cohort Norwegian Study on Coronary Heart Disease [NORCAD] with REC approval number: 2013/2324). All patients provided written consent.

### Targeted Metabolomics and Laboratory Measures

Blood samples were collected in tubes containing ethylenediamine tetra-acetic acid (EDTA) from non-fasting participants, kept on ice before centrifugation that occurred within 3 hours. Blood was drawn between 08:00 and 12:00. Patients were not routinely fasting. Samples were inspected during routine handling, and grossly hemolyzed samples were not used. Samples were aliquoted and stored at −80° C before cryopreservation prior to analyses.^16^ Trp, Kyn, HK, kynurenic acid (KA), anthranilic acid (AA), 3-hydroxyanthranilic acid (HAA), XA, and QA were measured by Bevital AS (http://www.bevital.no) using liquid chromatography-tandem mass spectrometry (LC-MS/MS) assay.^17^ Except for QA, all kynurenines were analyzed in 2007 after a median storage time of 7 years (interquartile range (IQR) 2.2). QA were analyzed in 2014 after a median storage time of 12 years (IQR 2.0). The limit of detection for Kyn, AA, KA, HK, HAA, and XA were between 0.5 nmol/L to 7 nmol/L and 0.4 µmol/L for Trp.^18^ Samples were randomized per plate with 88 samples per plate. Reported analytical coefficients of variation (CV) for the assay platform were low, with estimated within-day and between-day CVs ranging from 2.5% to 9.5% and 5.4% to 16.9%, respectively.^17^ The Kyn to Trp ratio (KTR) was calculated as Kyn (in nmol/L) divided by Trp (in µmol/L). Within-person stability stored 1, 2, and 3 years apart is good.^19^ However, HK, HAA can decrease and AA increase over time with improper sample handling such as longer storage at room temperature or storage at −25° C rather than −80° C.^20^ Authentic isotope-labeled internal standards were used for each analyte, and calibrators and in-house pooled plasma/serum quality control samples were run with each analytical batch to monitor assay performance within and between batches.

Creatinine was measured by the hospital laboratories. Estimated glomerular filtration rate (GFR) was calculated using the Chronic Kidney Disease Epidemiology Collaboration (CKD-EPI) formula. Diabetes was defined as self-reported, fasting glucose ⩾ 7.0 mmol/L or non-fasting glucose > 11.1 mmol/L. Serum cotinine level ⩾ 85nmol/L or self-reported smoking defined smoking status. TnT was measured using a high sensitivity assay on Modular E170 made by Roche diagnostics. The limit of detection was 3 ng/L and the upper 99th percentile 14 ng/L.^21^

### Statistics

Except for Trp, all kynurenines followed non-normal distributions, as did LVEF and TnT. These followed log-normal distributions and were thus log-transformed. Variables were inspected visually using histograms and Q-Q plots before and after transformation. Transformations were considered using Tukey’s ladder of powers, and non-linear terms (quadratic, cubic, and square-root forms) were screened by comparison of the Bayesian Information Criterion, with a decrease of 5 considered evidence of improved fit. As no clear improvement was found, metabolites were modeled in their log-transformed linear form before standardization (mean 0 and standard deviation [SD] 1) to facilitate comparison of effect sizes within the study. Missingness was low for all baseline covariates included in the analyses (<5%) and we did not suspect informative missingness.^22^ Missing covariate values were imputed using gender-specific means for normally distributed variables and gender-specific medians for non-normally distributed variables, to preserve sample size and statistical power. There were no missing kynurenine measurements among included participants. Cox proportional hazard analysis was used to estimate the associations with incident HF and incident AMI. The proportional hazards assumption was evaluated by inspection of Schoenfeld residuals. The model was adjusted for age, gender, current smoker, diabetes mellitus, hypertension, previous MI, BMI, GFR, TnT, baseline heart rate, and LVEF, all of which are known risk factors for HF.^23-27^ Medication variables were not included in the main model because baseline drug use in this cohort was considered strongly confounded by indication. As AMI is a major determinant of HF, we also compared the kynurenine-associated risk of incident AMI with that of incident HF, using an identical unadjusted and adjusted Cox regression. Previous MI was chosen over the number of vessels affected on coronary angiography as a model covariate because MI remained more associated with the outcome in the final model. Correlations between KP metabolites and baseline LVEF and TnT were examined using Spearman’s rho. Spearman correlations between storage time and metabolite concentrations were also examined and are shown in Supplemental Table 1. A *P*-value of .05, adjusted for multiple comparisons (9 metabolites tested in separate analyses per analytic approach) according to the Bonferroni correction (0.05/9) was considered significant (*P* < .006). All analyses and figures were done using Stata (StataCorp. 2023. *Stata Statistical Software: Release 18*. College Station, TX: StataCorp LLC).

## Results

### Study Participants

The baseline characteristics of the participants are summarized in Table 1. A total of 221 patients developed HF and 261 developed AMI during a median follow-up time of 7.5 years (IQR 2.2). Kaplan-Meier estimates of HF-free survival during follow-up are shown in Supplemental Figure 1, with yearly numbers at risk, events, and censoring presented in Supplemental Table 2. Patients with incident HF were older, had a lower percentage of smokers and a higher number with hypertension than patients who did not develop HF. Further, patients with incident HF and incident AMI had more coronary artery disease as assessed by baseline angiography, compared with patients who developed new AMI. Sixty-six patients overlapped in the groups, due to the occurrence of both HF and AMI in the same patients.

**Table 1.** Baseline Characteristics.

Clinical characteristics and metabolites	All (N = 3841)	HF (N = 221)	AMI (N = 261)
Age, y; mean (SD)	61.5 (10.4)	68.5 (9.6)	64.9 (10.5)
Male, N (%)	2743 (71.3)	162 (73.3)	198 (75.9)
Heart rate, beats per min	63.6 (11.8)	66.3 (12.6)	66.2 (12.6)
Body mass index, mean (SD)	26.7 (3.9)	27.3 (4.7)	26.8 (4.5)
Diabetes; N (%)	430 (11.2)	44 (19.9)	51 (19.5)
Current smoker, N (%)	1218 (31.7)	65 (29.4)	108 (41.4)
Hypertension, N (%)	1787 (46.5)	144 (65.2)	148 (56.7)
Previous MI, N (%)	1424 (36.3)	116 (52.5)	149 (57.1)
GFR, mL/min/1.73 m^2^, mean (SD)	88.5 (16.6)	79.2 (20.1)	82.6 (21.8)
Troponin T, ng/L, mdn (IQR)	4.0 (6.0)	10.0 (11.0)	9.0 (15.0)
Tryptophan, μmol/L, mdn (IQR)	70.2 (18.9)	68.3 (19.7)	68.4 (20.3)
Kynurenine, μmol/L, mdn (IQR)	1.66 (0.61)	1.79 (0.67)	1.75 (0.64)
Kynurenic acid, nmol/L, mdn (IQR)	47.6 (24.8)	51.1 (27.7)	51.5 (27.5)
Anthranilic acid, nmol/L, mdn (IQR)	14.3 (6.89)	15.7 (6.81)	15.6 (8.63)
3-hydroxykynurenine, nmol/L, mdn (IQR)	30.4 (15.4)	35.6 (18.3)	34.2 (18.5)
3-hydroxyanthranilic acid, nmol/L, mdn (IQR)	34.0 (19.2)	36.0 (20.4)	37.0 (23.5)
Xanthurenic acid, nmol/L, mdn (IQR)	14.3 (9.68)	13.8 (10.0)	13.8 (10.1)
Quinolinic acid, nmol/L, mdn (IQR)	392 (175)	487 (220)	438 (268)
Kynurenine:tryptophan ratio, mdn (IQR)	2.36 (0.89)	2.64 (1.15)	2.56 (1.28)
From coronary angiography			
Coronary artery stenosis, N (%)			
0	1010 (26.3)	38 (17.7)	28 (10.6)
1	907 (23.6)	38 (17.7)	56 (21.2)
2	863 (22.4)	46 (21.4)	65 (24.6)
3	1066 (27.7)	102 (43.3)	115 (43.6)
LVEF, mean (SD)	66.0 (8.8)	61.0 (10.3)	63.1 (10.0)

Abbreviations: AMI, acute myocardial infarction; GFR, glomerular filtration rate; HF, heart failure; IQR, interquartile range; LVEF, left ventricular ejection; mdn, median; MI, myocardial infarction; N, number of participants in listed category; SD, standard deviation.

Missing data (Age 0, male 0, heart rate 180, BMI 8, smoking 6, HT and previous MI 0, GFR 16, and troponin T 91).

### Kynurenine Concentrations and Correlation With Baseline Ejection Fraction or Troponin T

Baseline concentrations of all measured metabolites are shown in Table 1. Univariate comparisons of metabolite levels showed modest separation for both incident HF and AMI, with the largest AUCs observed for QA, HK, and KTR (Supplemental Table 3). The kynurenines showed low correlation with the baseline left ventricular ejection fraction as assessed by cine ventriculography, and only KTR was below the Bonferroni threshold (*P* = .006) with a weak effect size (*R* −.05). However, all the kynurenines were significantly correlated with baseline TnT (in order of *R*, QA 0.30, Kyn 0.27, KTR 0.25, HK 0.23, KA 0.23, AA 0.17, HAA 0.09, and XA 0.06, all *P* < .001). See Table 2.

**Table 2.** Correlations of Kynurenines With Baseline Measures of Ejection Fraction and Troponin T.

Metabolites	Left ventricular ejection fraction		Troponin T	
	*Rho* ^a^	*P*	*Rho* ^a^	*P*
Tryptophan	.03	.049	.01	.976
Kynurenine	−.03	.036	.27	<.001*
Kynurenic acid	−.02	.149	.23	<.001*
Anthranilic acid	.03	.083	.17	<.001*
3-hydroxykynurenine	−.02	.269	.23	<.001*
3-hydroxyanthranilic acid	−.01	.577	.09	<.001*
Xanthurenic acid	.01	.624	.06	<.001*
Quinolinic acid	−.03	.060	.30	<.001*
Kynurenine:tryptophan ratio	−.05	.002*	.25	<.001*

a Spearman Rho correlation coefficient.

* *P*-value < Bonferroni threshold: .006.

### Kynurenines and the Hazard of Heart Failure and Myocardial Infarction

Elevated plasma levels of HK, QA, and KTR were strongly associated with increased risk of HF and showed weaker associations with AMI. Trp was associated with reduced risk of HF (Table 3A).

**Table 3.** Metabolite Concentrations and the Hazard of Heart Failure and Acute Myocardial Infarction.

Metabolites	Heart failure			Acute myocardial infarction		
	HR	95% CI	*P*	HR	95% CI	*P*
(A) Unadjusted analyses						
Trp	0.82	0.73-0.94	.003*	0.85	0.76-0.96	.008
Kyn	1.37	1.20-1.56	<.001*	1.25	1.10-1.41	<.001*
KA	1.31	1.19-1.46	<.001*	1.26	1.14-1.39	<.001*
AA	1.22	1.09-1.38	.001	1.24	1.12-1.38	<.001*
HK	1.67	1.47-1.89	<.001*	1.33	1.18-1.50	<.001*
HAA	1.15	1.01-1.32	.038	1.16	1.02-1.31	.021
XA	0.99	0.87-1.13	.909	0.99	0.88-1.12	.884
QA	1.54	1.39-1.71	<.001*	1.34	1.20-1.48	<.001*
KTR	1.47	1.30-1.65	<.001*	1.34	1.20-1.50	<.001*
(B) Adjusted analyses						
Trp	0.91	0.80-1.03	.133	0.94	0.84-1.06	.320
Kyn	1.01	0.87-1.17	.947	1.09	0.95-1.24	.238
KA	1.02	0.88-1.17	.835	1.16	1.03-1.30	.013
AA	0.99	0.88-1.14	.999	1.13	1.01-1.28	.043
HK	1.31	1.07-1.46	<.001*	1.12	0.98-1.28	.086
HAA	1.07	0.94-1.22	.302	1.12	0.99-1.26	.071
XA	0.95	0.83-1.09	.504	0.94	0.84-1.07	.386
QA	1.21	1.05-1.40	.010	1.17	1.02-1.34	.028
KTR	1.09	0.95-1.26	.226	1.13	0.99-1.29	.064

Abbreviations: 95% CI, 95 percent confidence interval; AA, anthranilic acid; HAA, 3-hydroxyanthranilic acid; HK, 3-hydroxykynurenine; HR, hazard ratio; KA, kynurenic acid; KTR, kynurenine:tryptophan ratio; Kyn, kynurenine; QA, quinolinic acid; Trp, tryptophan; XA, xanthurenic acid.

(A) Univariate Cox regression with the listed predictors. HRs reported per 1 SD increase in the log-transformed variables. (B) Multivariate Cox regression as in A., adjusted for age, gender, current smoking, diabetes, hypertension, previous myocardial infarction, body mass index, glomerular filtration rate, troponin T, left ventricular ejection fraction, and baseline heart rate.

* *P*-value < Bonferroni threshold: .006.

**Figure 3.**
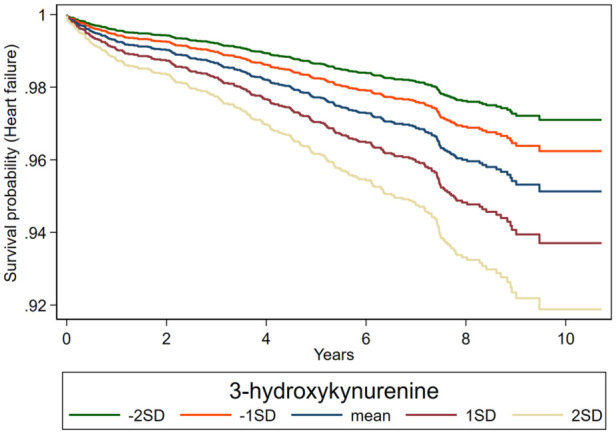
Heart failure free survival according to 3-hydroxykynurenine. Predictions of heart failure per one standard deviation of 3-hydroxykynurenine by Cox regression, adjusted for age, sex, current smoking, diabetes mellitus, hypertension, previous myocardial infarction, body-mass index, glomerular filtration rate, left ventricular ejection fraction, and heart rate.

In adjusted analyses, only HK remained associated with HF, after adjusting for multiple comparisons (Bonferroni threshold *P* = .006, Table 3B and Figure 3). The associations with the metabolites were non-significant for AMI in the adjusted model after Bonferroni correction.

Of the clinical factors considered as potential confounders, age, current smoking, higher BMI, diabetes mellitus, hypertension, TnT, and higher heart rate remained significantly associated with incident HF. Higher left ventricular ejection fraction was protective whereas previous MI, and eGFR did not reach significance in this multivariable model (Supplemental Table 4).

## Discussion

In this study we found that following adjustment for potential confounders, higher HK is associated with higher risk of incident HF. HK was not associated with risk of AMI, which may suggest that the observed association is more closely related to HF development than to ischemic events, although this should be interpreted cautiously. HK is related to apoptosis,^7^ but our data do not establish whether HK is causally involved in HF development or instead reflects underlying immune-metabolic activity.

At baseline, the concentrations of kynurenines were not associated with left ventricular function, apart from a very weak inverse association with KTR in patients without heart failure at baseline. However, there was a significant correlation between baseline TnT and kynurenines known to be associated with inflammation, where the strongest correlations were observed for QA, Kyn, and KTR.^6,28,29^ Whether the kynurenine pathway activation is a driver of disease progression or rather reflects a response to a shared milieu of low-grade myocardial injury and immune-metabolic activation remains to be established.

Our findings extend previous prospective work linking KP metabolites to future HF risk by examining a stable CAD cohort without HF at baseline and by comparing associations with incident HF and incident AMI within the same population. Consistent with our previous findings that elevated plasma levels of HK predict mortality in patients with established HF,^12^ HK was also associated with incident HF, which supports a consistent association between HK and HF across disease stages. HK has been shown to accelerate apoptosis in both mice and human cells.^7,30^ Elevated plasma levels of HK could theoretically play a role in apoptotic pathways and thus the development of myocardial dysfunction, but they could also reflect upstream processes not captured in our model, including chronic inflammation or compensatory activation of the KP. More broadly, KP activation may not be purely pathogenic, but could also represent an adaptive response to chronic inflammatory and metabolic stress, for example through immune regulation and support of de novo NAD+ synthesis. In that context, higher HK may reflect increased pathway flux rather than a directly harmful mediator. However, the kynurenines (Kyn, KTR, and QA) in particular associated with inflammation were less associated with HF than HK.^28,29^

Our findings support the relevance of altered immune-metabolic pathways in HF, while the direction and biological significance of these changes remain uncertain. Further epidemiological and mechanistic studies should be performed to validate our findings of association between HK and HF. Before interventional studies can be considered, experimental work is needed to clarify whether HK has a causal role in HF development, reflects an adaptive response, or is mainly a marker of underlying processes.

Strengths of this study include a large sample size, a systematic and robust measurement of coronary artery disease and LVEF at baseline, and a precise measurement of a large panel of kynurenines. Limitations of the study includes the post-study definition of HF based on registry-derived ICD-10 diagnoses and thus the low number of events relative to sample size, lack of systematic follow-up and lack of modern definitions of HF distinguishing those with reduced, mid-range and preserved LVEF at follow-up. This study did not include measurements of NT-proBNP, and there is thus a risk of undiagnosed HF patients not being included in the study. Together, the lack of NT-proBNP and contemporary HF diagnostic criteria may have led to under-detection or misclassification of milder HF cases. This would be expected mainly to reduce diagnostic sensitivity. Considering the lack of a priori power estimates and old definitions of HF, the study is explorative and hypothesis-generating and needs external validation. Further, an observational study cannot rule out that activation of the KP could be adaptive in chronic inflammation. We did however adjust for left ventricular ejection fraction and risk factors, which may partly reduce, but cannot eliminate, this concern. Residual confounding by medication use remains possible because baseline drug treatment in observational studies is often confounded by indication.^31^ The study did not standardize fasting, where one study found lower Trp, with high KA, HK, and PIC following 2 days of fasting.^32^ However, the time of day of sampling or procedures, which could affect fasting duration, was not planned according to disease severity. Storage time was weakly associated with KA and AA, but not with HK, suggesting that storage-related effects are unlikely to explain the main findings. Because missing data were mainly handled by complete-case analysis, some risks of selection bias cannot be excluded. As the KP metabolites are biologically correlated, the Bonferroni correction was conservative and may have increased type II error. Overall, our study should be considered exploratory, and our findings should be confirmed in prospective studies using modern and more precise definitions of HF.

Higher HK was associated with increased risk of HF but not AMI in patients with stable coronary artery disease, highlighting the importance of early metabolic changes in HF. These findings support a possible link between KP activity and future HF risk, but do not establish causality.

## Supplemental Material

sj-docx-1-try-10.1177_11786469261479281 – Supplemental material for The Kynurenine Metabolite 3-Hydroxykynurenine is Associated With Risk of Heart Failure in Patients With Predominantly Stable Coronary Artery Disease Supplemental material, sj-docx-1-try-10.1177_11786469261479281 for The Kynurenine Metabolite 3-Hydroxykynurenine is Associated With Risk of Heart Failure in Patients With Predominantly Stable Coronary Artery Disease by Anders Lund, Jan Erik Nordrehaug, Kjell-Christian Flo, Kyrre Hasås Toresen, Christian Alsing, Adrian McCann, Per Magne Ueland, Ottar Nygård and Lasse M. Giil in International Journal of Tryptophan Research
