# Supplementary material for: The Kynurenine Metabolite 3-Hydroxykynurenine is Associated With Risk of Heart Failure in Patients With Predominantly Stable Coronary Artery Disease
Source: Int J Tryptophan Res. 2026 Aug 18;19:11786469261479281. doi: 10.1177/11786469261479281 (PMC13487136; doi:10.1177/11786469261479281)
Supplement: Supplementary file 1 [file sj-docx-1-try-10.1177_11786469261479281.docx]

| **Supplementary table 1.** Spearman correlations with storage time | | |
| --- | --- | --- |
| Metabolite | Rho^a^ | p |
| Tryptophan | -0.01 | 0.887 |
| Kynurenine | 0.02 | 0.244 |
| Kynurenic acid | 0.05 | 0.003 |
| Anthranilic acid | 0.11 | <0.001 |
| 3-hydroxykynurenine | 0.02 | 0.260 |
| 3-hydroxyanthranilic acid | 0.02 | 0.236 |
| Xanthurenic acid | 0.01 | 0.515 |
| Quinolinic acid | -0.01 | 0.511 |
| Kynurenine:tryptophan ratio | 0.01 | 0.410 |
| ^a^ Spearman Rho correlation coefficient | | |

| **Supplementary table 2.** Numbers at risk, heart failure events, and censoring by year of follow-up | | | |
| --- | --- | --- | --- |
| Years in study | At risk | HF | Censored |
| 1 | 3767 | 15 | 42 |
| 2 | 3710 | 25 | 30 |
| 3 | 3655 | 30 | 42 |
| 4 | 3583 | 29 | 54 |
| 5 | 3500 | 27 | 147 |
| 6 | 3326 | 21 | 965 |
| 7 | 2340 | 27 | 780 |
| 8 | 1533 | 9 | 868 |
| 9 | 656 | 2 | 586 |
| 10 | 68 | 0 | 68 |
|  |  |  |  |

| **Supplementary Table 3.** Univariate associations | | | | | | |
| --- | --- | --- | --- | --- | --- | --- |
|  |  | **HF vs controls** | |  | **AMI vs controls** | |
|  |  | AUC | p |  | AUC | p |
| Age |  | 0.70 | <0.001 |  | 0.60 | <0.001 |
| Male |  | 0.51 | 0.462 |  | 0.53 | 0.074 |
| Heart rate |  | 0.58 | <0.001 |  | 0.57 | <0.001 |
| Body mass index |  | 0.54 | 0.052 |  | 0.50 | 0.996 |
| Diabetes |  | 0.55 | <0.001 |  | 0.54 | <0.001 |
| Current smoker |  | 0.51 | 0.631 |  | 0.55 | <0.001 |
| Hypertension |  | 0.60 | <0.001 |  | 0.56 | 0.001 |
| Previous MI |  | 0.60 | <0.001 |  | 0.61 | <0.001 |
| GFR |  | 0.36 | <0.001 |  | 0.42 | <0.001 |
| Coronary artery stenosis |  | 0.61 | <0.001 |  | 0.63 | <0.001 |
| LVEF |  | 0.33 | <0.001 |  | 0.42 | <0.001 |
| Troponin T |  | 0.72 | <0.001 |  | 0.65 | <0.001 |
| Tryptophan |  | 0.44 | 0.003 |  | 0.46 | 0.026 |
| Kynurenine |  | 0.58 | <0.001 |  | 0.56 | 0.001 |
| Kynurenic acid |  | 0.57 | <0.001 |  | 0.56 | <0.001 |
| Anthranilic acid |  | 0.57 | <0.001 |  | 0.57 | <0.001 |
| 3-hydroxykynurenine |  | 0.62 | <0.001 |  | 0.58 | <0.001 |
| 3-hydroxyanthranilic acid |  | 0.54 | 0.058 |  | 0.55 | 0.010 |
| Xanthurenic acid |  | 0.49 | 0.450 |  | 0.49 | 0.691 |
| Quinolinic acid |  | 0.65 | <0.001 |  | 0.59 | <0.001 |
| Kynurenine:tryptophan ratio |  | 0.62 | <0.001 |  | 0.58 | <0.001 |
| Abbreviations: AMI, acute myocardial infarction; AUC, area under the curve; HF, heart failure; IQR, interquartile range; GFR, glomerular filtration rate; LVEF, left ventricular ejection; MI, myocardial infarction; N, number of participants in listed category; SD, standard deviation. | | | | | | |

| **Supplementary Table 4.** Hazard ratios for potential confounders in Cox regression models with incident HF as outcome, each adjusted for 3-hydroxykynurenine. | | | | |
| --- | --- | --- | --- | --- |
|  |  | HR | 95% CI | p |
| Age |  | 2.08 | 1.71, 2.51 | <0.001* |
| Male |  | 1.08 | 0.80, 1.46 | 0.601 |
| Current smoker |  | 1.41 | 1.05, 1.89 | 0.021* |
| Diabetes mellitus |  | 1.45 | 1.06, 1.99 | 0.022* |
| Hypertension |  | 1.44 | 1.09, 1.40 | 0.009* |
| Previous MI |  | 1.14 | 0.88, 1.49 | 0.314 |
| BMI |  | 1.23 | 1.09, 1.39 | 0.001* |
| GFR |  | 1.10 | 0.94, 1.29 | 0.232 |
| TnT |  | 1.30 | 1.16, 1.45 | <0.001* |
| LVEF |  | 0.59 | 0.52, 0.67 | <0.001* |
| Heart rate |  | 1.31 | 1.13, 1.51 | 0.002* |
| HRs per 1 standard deviation in each continuous variable (TnT and LVEF log-transformed) or per category versus reference for binary variables.  Abbreviations: BMI, body-mass index; GFR, glomerular filtration rate; HR, hazard ratio; LVEF, left ventricular ejection fraction; MI, myocardial infarction; TnT, troponin T.  * p-value < 0.05 | | | | |

**Supplementary Figure 1. Kaplan-Meier curve for incident heart failure during follow-up.** Kaplan-Meier estimates of heart failure-free survival in the study population over follow-up time. The solid line shows the estimated survival function, and the shaded area indicates the 95% confidence interval. Follow-up time is shown in years from baseline. Yearly numbers at risk, events, and censoring are presented in Supplementary Table 2.
